# Association of composite dietary antioxidant index with prevalence of stroke: insights from NHANES 1999-2018

**DOI:** 10.3389/fimmu.2024.1306059

**Published:** 2024-03-08

**Authors:** Tian-Qi Teng, Jing Liu, Fang-Fang Hu, Qing-Qing Li, Zhen-Zhu Hu, Yu Shi

**Affiliations:** ^1^ Department of Cardiology, Xu Zhou New Health Geriatric Hospital, Xuzhou, Jiangsu, China; ^2^ Department of Neurology, Xu Zhou New Health Geriatric Hospital, Xuzhou, Jiangsu, China; ^3^ The Affiliated XuZhou Hospital of Jiangsu University, Xuzhou, Jiangsu, China

**Keywords:** CDAI, stroke, NHANES, cross-sectional study, RCS

## Abstract

**Background:**

There is a growing acknowledgment of the potential influence of antioxidative effects resulting from dietary decisions on the occurrence of stroke. The objective of this study was to elucidate the correlation between the composite dietary antioxidant index (CDAI) and the incidence of stroke in the general population of the United States.

**Methods:**

We gathered cross-sectional data encompassing 40,320 participants from the National Health and Nutrition Examination Survey (NHANES) spanning the years 1999 to 2018. Employing weighted multivariate logistic regression, we assessed the correlation between CDAI and stroke, while also investigating potential nonlinear relationships through restricted cubic spline (RCS) regression. Further, the intake of CDAI components were then incorporated into a predictive nomogram model, subsequently evaluated for its discriminatory prowess in stroke risk assessment using the receiver operating characteristic (ROC) curve.

**Results:**

Post-adjustment for confounding variables, we found that higher CDAI score were associated with a decreased risk of stroke, the odds ratio (OR) [95% CI] of CDAI associating with prevalence was 0.96 [0.94-0.98] (P< 0.001). Moreover, the adjusted OR [95% CI] for stroke across ascending CDAI quartiles stood at 0.90 [0.74-1.09], 0.74 [0.60-0.91], and 0.61 [0.50-0.76] compared to the reference quartile, respectively. The RCS analysis indicated a nonlinear yet negative correlation between CDAI and stroke. The nomogram model, constructed based the intake of antioxidants, exhibited a significant predictive capacity for stroke risk, boasting an area under the curve (AUC) of 77.4% (76.3%–78.5%).

**Conclusion:**

Our investigation ascertained a nonlinear negative relationship between CDAI and stroke within the broader American population. However, given the inherent limitations of the cross-sectional design, further comprehensive research is imperative to establish the causative nature of this association.

## Introduction

Stroke is a devastating cerebrovascular event that continues to pose a significant public health challenge worldwide (1). According to the World Health Organization (WHO) estimates, there were approximately 13.7 million new cases of stroke worldwide each year (2, 3). As one of the leading causes of disability and mortality, understanding the multifactorial nature of stroke and identifying modifiable risk factors are essential for effective prevention and management strategies (4, 5). Hypertension is a significant risk factor for both ischemic stroke and hemorrhagic stroke, other contributing factors include diabetes, smoking, obesity, sedentary lifestyle, and certain medical conditions can also promote blood clot formation (6, 7). Identifying and understanding the causes of stroke are crucial in adopting preventive measures and promoting better overall health.

Among the various contributors to stroke, oxidative stress has emerged as a critical underlying mechanism. Oxidative stress, resulting from an imbalance between reactive oxygen species (ROS) production and the body’s ability to neutralize them with antioxidants, has been linked to endothelial dysfunction, inflammation, and ultimately, vascular damage (8). Free radicals are highly reactive molecules that can cause damage to cells, proteins, and DNA (9). In the context of stroke, which is a medical emergency involving the sudden interruption of blood flow to the brain, oxidative stress becomes a major contributor to the cascade of events leading to brain injury (10). During a stroke, the lack of oxygen and nutrients triggers the production of harmful free radicals, exacerbating the damage to brain tissue and potentially worsening the neurological outcomes for affected individuals (11).

Dietary habits have garnered significant attention due to their direct influence on overall health. Extensive research over the years has established a compelling association between dietary choices and the incidence of stroke (12, 13). Composite Dietary Antioxidant Index (CDAI) is a novel and comprehensive tool designed to assess the combined antioxidant intake from a diverse range of dietary sources (14). The CDAI amalgamates data from multiple antioxidant-rich foods and provides a standardized metric for quantifying antioxidant consumption in individuals and populations. CDAI has been demonstrated positive impacts on various chronic diseases, including blood pressure, diabetes, arthritis, and infertility-related conditions (15–17). By utilizing the CDAI, researchers, clinicians, and public health professionals can gain a deeper understanding of the intricate interplay between dietary antioxidants and their impact on human health, ultimately paving the way for more targeted and personalized dietary recommendations.

However, to the best of knowledge, there have been no study investigated the association between CDAI and the prevalence of stroke. In this study, we present the results of an in-depth investigation into the potential protective effects of dietary antioxidants against stroke. By calculating CDAI in the present study, we aimed to capture the synergistic effects of various antioxidants present in the diet, going beyond the analysis of individual antioxidants alone.

## Methods

### Study population

**Figure 1:**
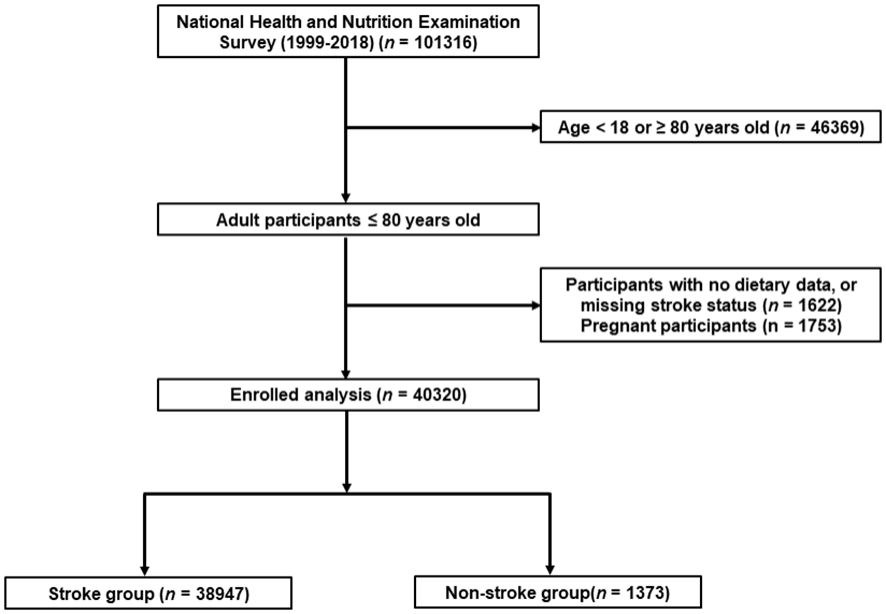
A detailed flow chart of participant recruitment.

NHANES, a nationwide initiative established by the National Center for Health Statistics (NCHS), is primarily dedicated to assessing the health and nutritional status of noninstitutionalized civilians in the United States through biennial assessments. Its primary objective is to gain a comprehensive understanding of contemporary disease patterns and offer insights for shaping public health policies. The entirety of NHANES data is accessible to the general public and can be freely obtained from: https://www.cdc.gov/nchs/nhanes/index.htm. For this particular research, data from 101,316 participants across ten consecutive NHANES cycles (1999–2018) were initially gathered. Specific exclusion criteria were applied as follows (1): participants aged below 18 or above 80 years (n = 46,369) (2); pregnant participants (n = 1,753); (3) individuals lacking pertinent information on dietary intake or stroke status (n = 1,622). Following meticulous data screening, a total of 40,320 participants were ultimately chosen for subsequent analyses. In the eventually enrolled participants, there is no individual with daily caloric intake above 4500 and less than 700 kcal. A comprehensive flowchart illustrating the recruitment process for study participants can be found in **Figure 1**.

### Assessment of CDAI

While maintaining the same meaning and ensuring the repetition rate stays below 30%: The NHANES dataset recorded the dietary intake of each participant through a 24-hour recall interview. The initial interview was conducted face-to-face, followed by a telephone interview within 3 to 10 days. To determine antioxidant, micronutrient, and total energy intake, the United States Department of Agriculture’s Food and Nutrient Database for Dietary Studies was employed. Additionally, the questionnaire assessed the use of dietary supplements in the past month, encompassing frequency, dosage, and duration of consumption. Standardization of six antioxidants (manganese, selenium, zinc, vitamins A, C, and E) involved subtracting the mean and dividing by the standard deviation. In our study, we employed the energy-adjusted quantities of the six nutrients when calculating the dietary antioxidant index. The cumulative standardized consumption of these was utilized to calculate the CDAI (18). CDAI was calculated by the following formula:


$$\text{CDAI}=\sum_{i=1}^{6}(Xi-\text{μ}i)/Si$$

Xi represented the everyday antioxidant intake i; µi represented the mean Xi of the whole cohort for antioxidants i; Si represented the standard deviation (SD) for µ i.

### Assessment of stroke

The identification of a stroke was established based on individuals’ self-reported prior diagnosis by a medical practitioner during a face-to-face interview. Individuals responding affirmatively to the query, “Have you ever been informed by a physician or healthcare provider that you experienced a stroke?” were categorized as having had a stroke. It is important to note that relying on self-reported measures can be susceptible to memory bias, which might impact the interpretation of the collected information. Additionally, even though the NHANES database lacks specific details regarding the type of stroke, it is plausible that the majority of participants identified as having had a stroke in this study likely experienced ischemic strokes. This assumption is informed by the higher prevalence of ischemic strokes among stroke cases and their stronger association with chronic low-grade inflammatory conditions (12, 19).

### Covariates

Drawing from previous literature and biological considerations, we aimed to encompass an extensive range of covariates known to confound stroke outcomes. Standardized questionnaires and face-to-face interviews were utilized to gather demographic data, including age, gender, race/ethnicity, education level, smoking history, and alcohol consumption. The comprehensive medical examinations were meticulously conducted by experienced healthcare professionals at the MEC. Ethnicity was stratified into five distinct categories: non-Hispanic White, non-Hispanic Black, other Hispanic, Mexican American, and other racial groups. Educational attainment was classified as below high school, high school, or above high school. Smokers were identified as individuals who had smoked over 100 cigarettes in their lifetime, irrespective of their current smoking status at the time of the interview. Meanwhile, alcohol consumption was defined as consuming at least 12 drinks within the year preceding the survey. Body mass index (BMI), calculated by dividing weight in kilograms (kg) by height in meters squared (m²), served as the prevalent measure for assessing overweight and obesity. A BMI exceeding 25 and 30 was considered diagnostic for overweight and obesity respectively (20). Experienced clinicians, following standardized protocols, gauged systolic/diastolic blood pressure (SBP/DBP). Three consecutive readings, spaced at half-minute intervals, were averaged to yield the final blood pressure value. Laboratory tests, adhering to standardized procedures, were employed to determine fasting blood glucose (FBG), serum concentrations of triglycerides (TG), total cholesterol (TC), low-density lipoprotein cholesterol (LDL-C), high-density lipoprotein cholesterol (HDL-C), hemoglobin (Hb), glycated hemoglobin (HbA1c), red blood cell (RBC) counts, white blood cell (WBC) counts, neutrophil counts, monocyte counts, lymphocyte counts, and platelet counts. For estimating the glomerular filtration rate (eGFR), NHANES researchers employed a formula developed by the Chronic Kidney Disease Epidemiology Collaboration (CKD-EPI), which incorporated variables such as age, gender, race/ethnicity, and serum creatinine (SCr) to accommodate different populations. Considering hypertension’s pivotal role as a potential stroke precursor, it was crucial to mitigate its potential confounding influence. Hypertension was defined based on self-reported physician diagnosis or measured blood pressure during physical examinations. Participants meeting at least one of the following criteria were classified as having hypertension (1): Average systolic blood pressure (SBP) ≥ 140 mmHg; (2) Average diastolic blood pressure (DBP) ≥ 90 mmHg; (3) Self-reported hypertension diagnosis; (4) Current use of antihypertensive medications. Furthermore, diabetes represented another significant confounder with potential implications for stroke. Individuals with a previous physician or health professional diagnosis of diabetes were categorized as having diagnosed diabetes. Those lacking a diagnosed diabetes history but exhibiting HbA1c levels of 6.5% (47.5 mmol/mol) or higher, fasting plasma glucose (FPG) levels of 126 mg/dL (7.0 mmol/L) or higher, or 2-hour oral glucose tolerance test (OGTT) plasma glucose levels of 200 mg/dL or higher (11.1 mmol/L), as determined by laboratory tests, were classified as having undiagnosed diabetes. Both individuals with diagnosed and undiagnosed diabetes were collectively considered as diabetic patients.

### Statistical methods

Due to the intricate sampling methodologies employed in the NHANES survey, our analytical techniques considered sample weights specific to distinct research periods. This approach ensured precise calculations of health-related statistics. Weighted means and standard deviations (SD) represented continuous variables, while categorical variables were conveyed as frequencies and proportions. To discern variations in baseline traits between participants with and without stroke, student’s t-test and chi-square test were utilized for continuous and categorical variables, respectively. The CDAI scores were evenly stratified into four quartiles, with the initial quartile (Q1) as the reference. For the assessment of the association between CDAI and stroke, several multivariate logistic regression models were employed, including an unadjusted model, and two adjusted models (Model I and Model II). Model I incorporated adjustments for age, sex, and race/ethnicity, while Model II further accounted for educational level, smoking status, alcohol consumption, BMI, diabetes, and hypertension. There was no multicollinearity in the final model. The potential non-linear relationship between CDAI and stroke was examined using restricted cubic spline (RCS) regression with three knots (10th, 50th, and 90th percentiles). Subgroup analyses based on age, sex, race/ethnicity, BMI, smoking status, alcohol consumption, diabetes, and hypertension were performed to assess the stability of the CDAI-stroke association across various subgroups. Furthermore, a predictive nomogram model was developed using key stroke-related variables, and its predictive capability was assessed using the receiver operating characteristic (ROC) curve. Statistical analyses were carried out using R software version 4.1.6 (http://www.R-project.org, The R Foundation, Vienna, Austria), with statistical significance set at a two-tailed P-value< 0.05.

## Results

### Characteristics of the study population

In the present study, a total of 101316 participants of NHANES from 1999 to 2018 were initially included. According the inclusion and exclusion criteria, 40320 adults with dietary recall information were eventually enrolled, representing 17.6 million residents in the US (**Figure 1**). The mean age of the whole study population was 45.9 years, and slightly less half (49.29%) of the participants were male. Of them, 1372 participants (3.4%) had a history of stroke. Participants who had a history of stroke were generally older (60.2 years vs 45.5 years), and more likely to have hypertension (77.02% vs 35.32%), diabetes (6.61% vs 5.84%), and cardiovascular diseases, including coronary artery disease (17.36% vs 2.70%), angina (12.21% vs 1.89%), heart failure (15.86% vs 1.67%), and heart attack (20.89% vs 2.64%). Detailed demographic and clinical characteristics were showed in **Table 1**. **Supplementary Table S1** showed the demographic and clinical characteristics of the study population grouped by CDAI quantiles. The mean CDAI of the study population was 0.78, individuals who have a stroke history also have lower CDAI (-0.29 vs 0.81). Moreover, the intake of vitamin A (552.74 mg vs 627.11 mg), vitamin C (74.97 mg vs 83.43 mg), vitamin E (6.85 mg vs 8.39 mg), zinc (10.28 mg vs 11.95 mg), selenium (98.43 mcg vs 115.28 mcg), and carotenoid (8489.61 mcg vs 9802.70 mcg) were all lower in the stroke group (**Table 2**).

**Table 1:** Clinical characteristics of the study population grouped by stroke.

Variables	Overall (n = 40320)	Non- Stroke (n = 38947)	Stroke (n = 1373)	*P* value
**Age, years**				< 0.001***
**18-40 years**	39.83 [38.30, 41.35]	40.62 [39.57, 41.67]	8.97 [6.86, 11.09]	
**40-60 years**	39.63 [37.80, 41.46]	39.74 [38.91, 40.58]	35.09 [31.67, 38.51]	
**> 60 years**	20.55 [19.42, 21.67]	19.64 [18.89, 20.38]	55.93 [52.50, 59.37]	
**Sex-male, %**	49.29 [47.46, 51.13]	49.40 [48.90, 49.89]	45.34 [42.04, 48.65]	0.02
**Race, %**				< 0.001***
**Non-Hispanic White**	68.15 [63.80, 72.50]	68.19 [66.03, 70.35]	66.48 [62.66, 70.30]	
**Non-Hispanic Black**	11.23 [10.24, 12.22]	11.08 [9.92, 12.24]	16.96 [14.51, 19.41]	
**Mexican American**	8.29 [7.27, 9.30]	8.36 [7.24, 9.49]	5.32 [3.93, 6.71]	
**Other Hispanic**	5.44 [4.68, 6.21]	5.50 [4.70, 6.29]	3.35 [2.11, 4.60]	
**Other**	6.90 [6.33, 7.47]	6.87 [6.26, 7.48]	7.89 [5.72, 10.05]	
**Smoking, %**	22.02 [20.91, 23.12]	21.82 [21.04, 22.61]	29.74 [26.59, 32.89]	< 0.001***
**Drinking, %**	83.31 [79.97, 86.65]	89.40 [88.49, 90.31]	85.39 [82.56, 88.23]	< 0.001***
**Education level, %**				< 0.001***
**Below high school**	5.22 [4.81, 5.63]	5.10 [4.69, 5.51]	9.94 [7.85, 12.03]	
**High school**	34.51 [32.72, 36.31]	34.21 [32.98, 35.44]	47.14 [43.70, 50.59]	
**Above high school**	60.21 [57.57, 62.85]	60.69 [59.30, 62.08]	42.92 [39.27, 46.56]	
**SBP, mmHg**	121.49 [121.18, 121.81]	121.28 [120.97, 121.59]	129.86 [128.28, 131.44]	< 0.001***
**DBP, mmHg**	71.49 [71.18, 71.80]	71.50 [71.19, 71.81]	70.98 [70.06, 71.91]	0.26
**DM, %**	5.86 [5.82, 5.89]	5.84 [5.80, 5.87]	6.61 [6.37, 6.84]	< 0.001***
**FBG, mmol/L**	5.58 [5.57, 5.60]	5.57 [5.55, 5.58]	6.05 [5.96, 6.14]	< 0.001***
**HbA1c, %**	95.28 [94.76, 95.81]	95.74 [95.21, 96.26]	77.47 [75.72, 79.22]	< 0.001***
**eGFR, ml/min/1.73m^2^**	12.67 [12.05, 13.29]	12.08 [11.59, 12.57]	35.52 [32.17, 38.87]	< 0.001***
**TG, mmol/L**	1.49 [1.46, 1.52]	1.48 [1.45, 1.51]	1.67 [1.55, 1.79]	0.003**
**TC, mmol/L**	5.07 [5.05, 5.09]	5.07 [5.05, 5.09]	4.93 [4.85, 5.02]	0.003**
**LDL-C, mmol/L**	2.98 [2.96, 3.00]	2.99 [2.97, 3.01]	2.85 [2.75, 2.95]	0.002**
**HDL-C, mmol/L**	1.37 [1.36, 1.38]	1.37 [1.37, 1.38]	1.32 [1.29, 1.36]	0.01*
**RBC, ×10^9^/L**	4.73 [4.71, 4.74]	4.73 [4.72, 4.74]	4.59 [4.54, 4.63]	< 0.001***
**WBC, ×10^9^/L**	7.26 [7.21, 7.30]	7.25 [7.20, 7.30]	7.52 [7.38, 7.66]	< 0.001***
**NE, ×10^9^/L**	4.30 [4.26, 4.33]	4.29 [4.26, 4.32]	4.59 [4.47, 4.70]	< 0.001***
**Monocyte, ×10^9^/L**	0.56 [0.55, 0.56]	0.56 [0.55, 0.56]	0.59 [0.57, 0.61]	< 0.001***
**LY, ×10^9^/L**	2.15 [2.14, 2.17]	2.15 [2.14, 2.17]	2.07 [2.02, 2.12]	0.001**
**PLT, ×10^6^/L**	253.98 [252.54, 255.42]	254.14 [252.71, 255.57]	247.68 [241.20, 254.15]	0.04*
**Hemoglobin, g/L**	14.35 [14.31, 14.40]	14.36 [14.32, 14.41]	13.98 [13.86, 14.11]	< 0.001***
**CHD, %**	3.06 [2.76, 3.36]	2.70 [2.43, 2.97]	17.36 [14.64, 20.08]	< 0.001***
**Angina, %**	2.14 [1.90, 2.38]	1.89 [1.69, 2.08]	12.21 [9.62, 14.80]	< 0.001***
**HF, %**	2.02 [1.83, 2.21]	1.67 [1.51, 1.83]	15.86 [13.53, 18.19]	< 0.001***
**Hypertension, %**	36.36 [34.82, 37.91]	35.32 [34.46, 36.18]	77.02 [74.12, 79.93]	< 0.001***
**Heart attack, %**	3.10 [2.82, 3.38]	2.64 [2.42, 2.87]	20.89 [17.84, 23.94]	< 0.001***

Continuous variables are presented as the mean [95% CI], category variables are presented as the proportion [95% CI]. CI, confidence interval; SBP, systolic blood pressure; DBP, diastolic blood pressure; DM, diabetes; FBG, fasting blood glucose; HbA1c, glycated hemoglobin; eGFR, estimated glomerular filtration rate; BMI, body mass index; WC, waist circumference; TG, triglycerides; TC, total cholesterol; LDL-C, low-density lipoprotein cholesterol; HDL-C, high-density lipoprotein cholesterol; RBC, red blood cells; WBC, white blood cells; NE, neutrophils; LY, lymphocytes; PLT, platelets; CHD, coronary artery disease; HF, heart failure;. * P value<0.05, ** P value<0.01, *** P value<0.001.

**Table 2:** Comparison of CDAI and the intake of its components between stroke and non-stroke group.

Variables	Overall (n = 40320)	Non- Stroke (n = 38947)	Stroke (n = 1373)	*P* value
**CDAI**	0.78 [0.71, 0.86]	0.81 [0.73, 0.89]	-0.29 [-0.55, -0.03]	< 0.001***
**Vitamin A, mcg**	625.25 [612.82, 637.68]	627.11 [614.58, 639.64]	552.74 [515.19, 590.30]	<0.001***
**Vitamin C, mg**	83.22 [81.42, 85.02]	83.43 [81.63, 85.23]	74.97 [68.09, 81.85]	0.02*
**Vitamin E, mg**	8.35 [8.23, 8.48]	8.39 [8.27, 8.51]	6.85 [6.51, 7.19]	< 0.001***
**Zinc, mg**	11.91 [11.78, 12.04]	11.95 [11.82, 12.08]	10.28 [9.83, 10.74]	< 0.001***
**Selenium, mcg**	114.77 [113.81, 115.73]	115.28 [114.33, 116.23]	94.83 [90.51, 99.14]	< 0.001***
**Carotenoid, mcg**	9769.83 [9531.95, 10007.71]	9802.70 [9566.26, 10039.14]	8489.61 [7606.96, 9372.25]	0.003**

Data were presented as the mean and 95% confidence interval. CDAI, composite dietary antioxidant index. *** P value<0.001, ** P value<0.01, * P value<0.05.

### Associations of CDAI and its components with prevalence of stroke

**Figure 2:**
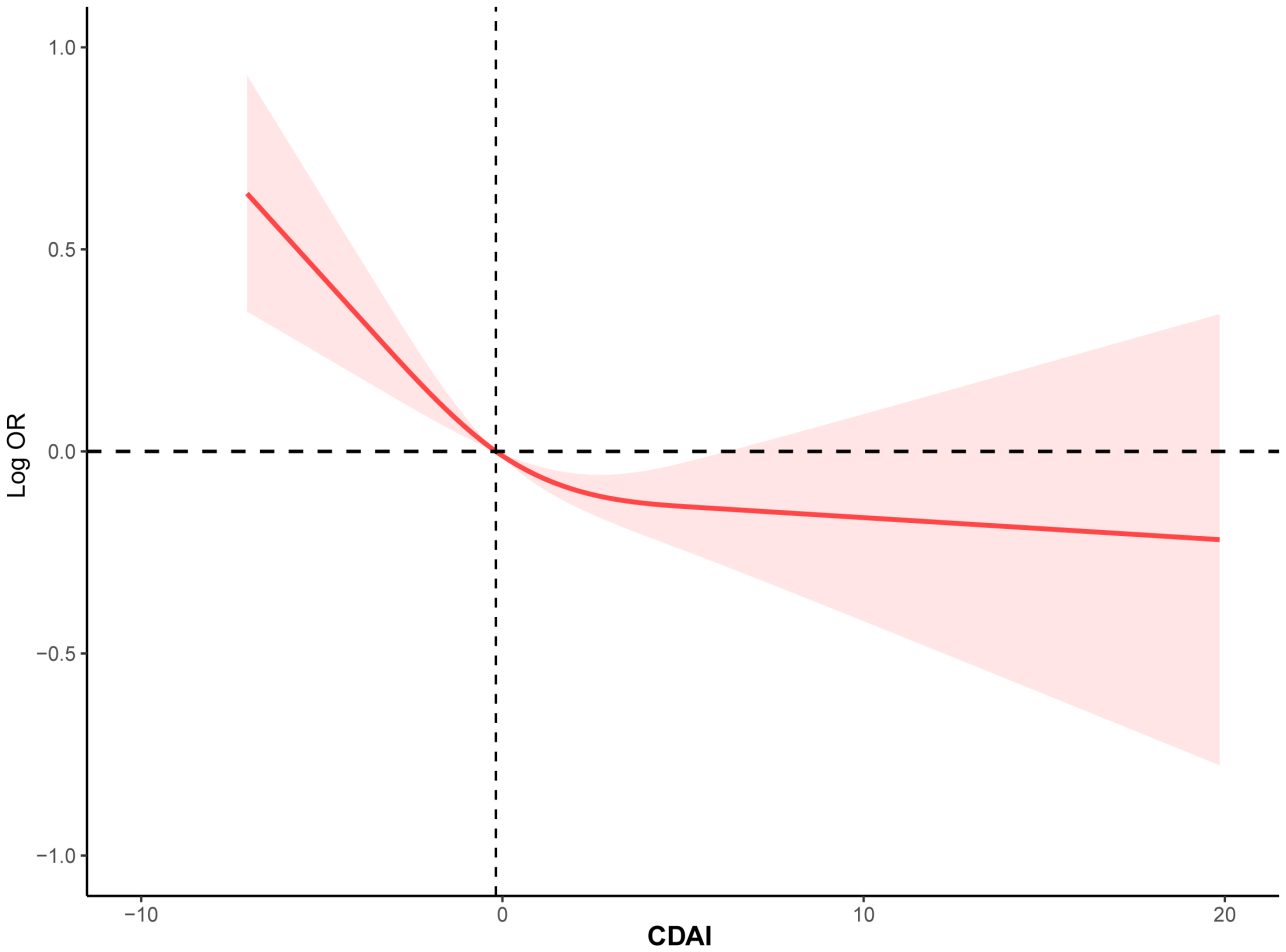
The RCS curve of the association between CDAI and stroke among all the study participants. RCS regression was adjusted for age, sex, race, education levels, smoking, drinking, hypertension, and DM. RCS, restricted cubic spline; CDAI, composite dietary antioxidant index; DM, diabetes; OR, odds ratio.

**Figure 3:**
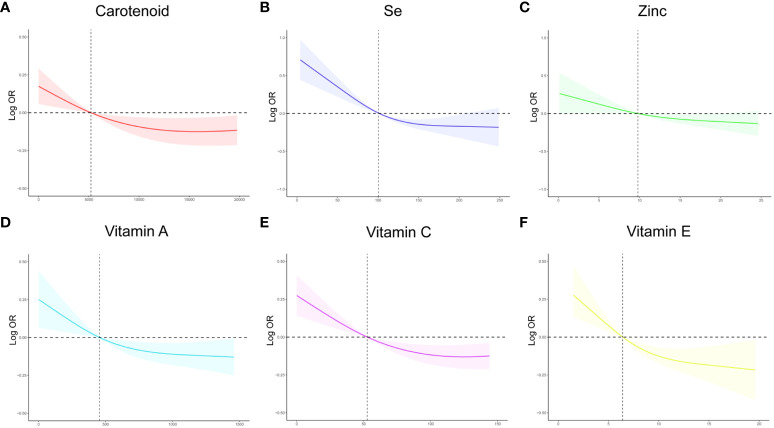
The RCS curves of the association between CDAI components and stroke among all the study participants. **(A)** The RCS curve of the association between carotenoid and stroke; **(B)** The RCS curve of the association between selenium and stroke; **(C)** The RCS curve of the association between zinc and stroke; **(D)** The RCS curve of the association between Vitamin A and stroke; **(E)** The RCS curve of the association between Vitamin C and stroke; **(F)** The RCS curve of the association between Vitamin E and stroke. RCS regression was adjusted for age, sex,race, education levels, smoking, drinking, hypertension, and DM. RCS, restricted cubic spline; CDAI, composite dietary antioxidant index; DM, diabetes; Se, selenium; OR, odds ratio.

To investigate the potential relationship between dietary antioxidant intake and stroke prevalence, we performed a weighted multivariate analysis adjusted for age, gender, ethnicity, education levels, smoking, drinking, hypertension, and DM. A statistically significant association was observed between CDAI and stroke prevalence before and after adjusting for covariables, suggesting that higher CDAI scores, as a continuous variable, were associated with a decreased risk of stroke, the odds ratio [95% confidence interval] of CDAI associating with prevalence was 0.96 [0.94-0.98] (P< 0.001) (**Table 3**). Additionally, based on the CDAI, all participants were evenly divided into quartiles, we also observed that the prevalence of stroke was significantly lower in participants with higher CDAI (**Table 3**). We also conducted RCS analysis to explore the relationships between the CDAI and its individual components with the prevalence of stroke. The RCS analysis revealed a significant association between CDAI and stroke prevalence, with a notable non-linear trend (P for non-linear trend< 0.001). As CDAI scores increased, the odds of stroke exhibited a gradual decline up to a certain threshold. Beyond the inflection point (3.66), the protective effect seemed to stabilize, suggesting a potential saturation effect at higher CDAI levels (**Figure 2**). Furthermore, we investigated the associations of each individual component of the CDAI with stroke prevalence using RCS analysis. Interestingly, certain combinations of antioxidants appeared to have synergistic effects, strengthening the overall protective association with stroke prevalence. Of note, the six component antioxidants of CDAI, including vitamin A, vitamin C, vitamin E, zinc, selenium, and carotenoid, all showed antagonistic effects, leading to an attenuated association with the risk of stroke (**Figure 3**).

**Table 3:** Weighted logistic regression analysis on the association between CDAI and stroke.

	Non-adjusted model		Model I		Model II	
	OR [95% CI]	*P* value	OR [95% CI]	*P* value	OR [95% CI]	*P* value
**Continuous CDAI**	0.92 [0.90, 0.94]	<0.001***	0.94 [0.91, 0.96]	<0.001***	0.98 [0.95, 0.99]	<0.01**
**CDAI-Q1**	Reference	–	Reference	–	Reference	–
**CDAI-Q2**	0.77 [0.65, 0.92]	0.004**	0.79 [0.66, 0.94]	0.01*	0.93 [0.76, 1.13]	0.29
**CDAI-Q3**	0.57 [0.47, 0.69]	<0.001***	0.61 [0.49, 0.74]	<0.001***	0.81 [0.72, 0.98]	0.01*
**CDAI-Q4**	0.42 [0.34, 0.51]	<0.001***	0.49 [0.40, 0.60]	<0.001***	0.72 [0.66, 0.86]	<0.001***

Data are presented as OR (95% CI). Model I adjusted for age, sex, and race/ethnicity. Model II adjusted for age, sex, race, education levels, smoking, drinking, energy intake, body mass index, physical activity, hypertension, and DM. *** P value<0.001, ** P value<0.01, * P value<0.05.

### Subgroup analysis on the association of CDAI and the prevalence of stroke among different populations

**Figure 4:**
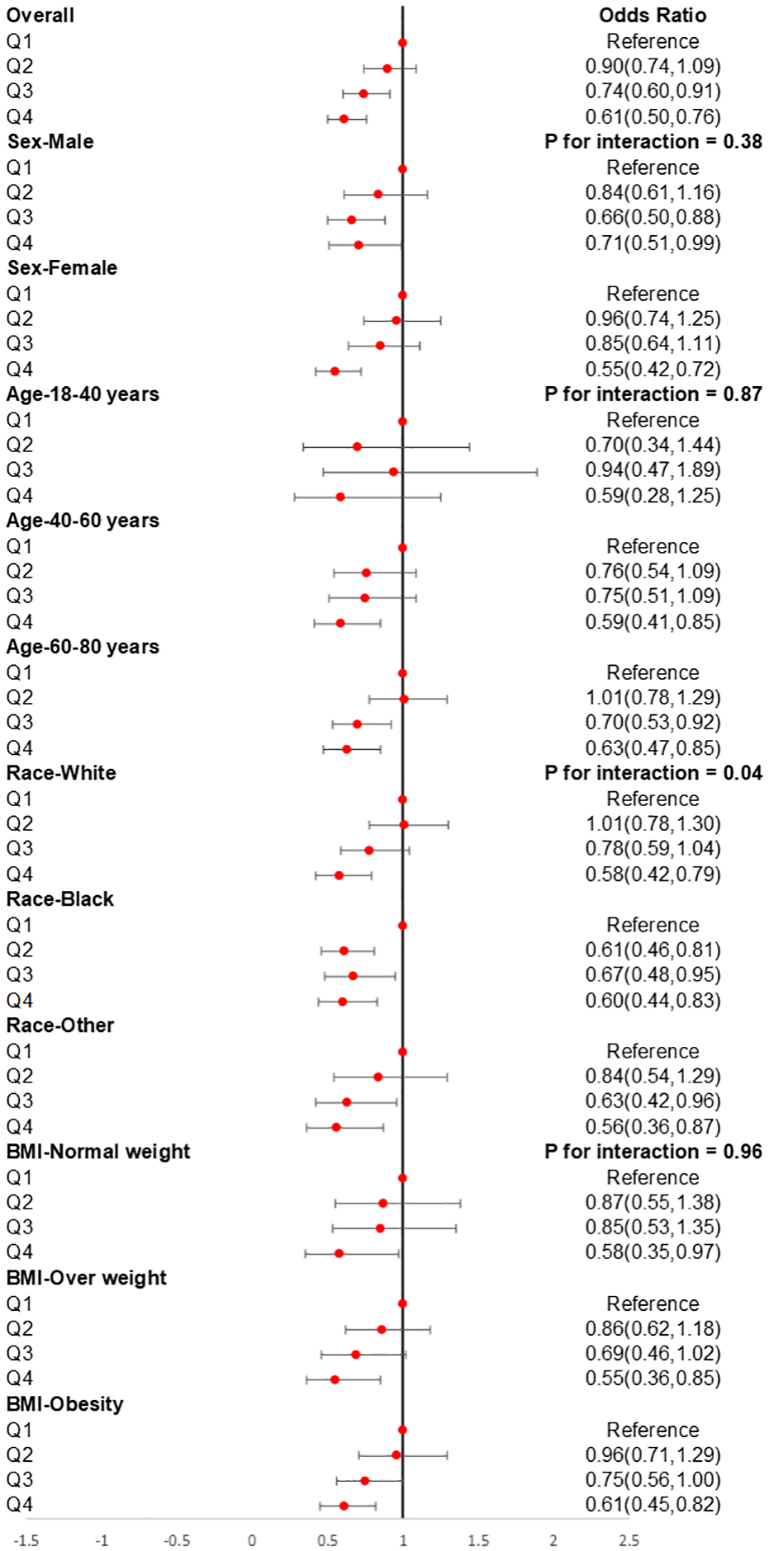
Subgroups analyses for the association between CDAI and stroke. Analyses were stratified by sex (male and female), age (≤ 40 years, 40–60 years, and ≥ 60 years), race/ethnicity (Black, White, and others), and BMI (normal weight, overweight, and obesity). Logistic regression analyses were adjusted for age, sex, race, education levels, smoking, drinking, hypertension, and DM. CDAI, composite dietary antioxidant index; DM, diabetes; OR, odds ratio.

**Figure 5:**
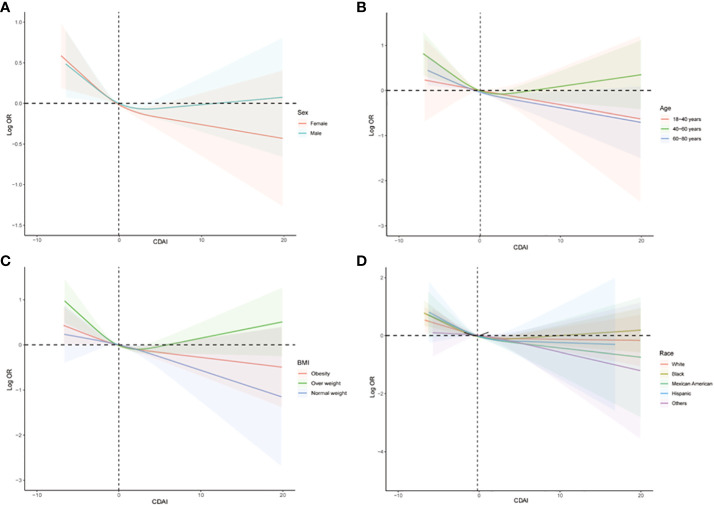
Subgroups RCS analyses for the association between CDAI and stroke stratified by **(A)** sex (male and female), **(B)** age (≤ 40 years, 40–60 years, and ≥ 60 years), and **(C)** BMI (normal weight, overweight, and obesity), and **(D)** race/ethnicity (Black, White, and others). RCS regression was adjusted for age, sex, race, education levels, smoking, drinking, hypertension, and DM. RCS, restricted cubic spline; CDAI, composite dietary antioxidant index; DM, diabetes; OR, odds ratio.

The subgroup analysis was also conducted to investigate the association between CDAI and the prevalence of stroke in different populations. As shown in **Figure 4**, we found that among participants of different genders, age groups, races, and levels of obesity, higher CDAI always associated with lower risk of stroke, indicating that the conclusion drawn in the present study is stable. It is noteworthy that in the subgroup analysis of different racial groups, we found that increasing the intake of antioxidants may have a greater benefit for black individuals (P for interaction = 0.04). We also carried out subgroup RCS analysis and found that after the inflection point, further elevating CDAI may lead to greater benefits for female participants, participants aged between 18-40 and 60-80 years, as well as participants with both obesity and normal body weight (**Figure 5**).

### Nomogram model for predicting stroke based on the intake of components of CDAI

**Figure 6:**
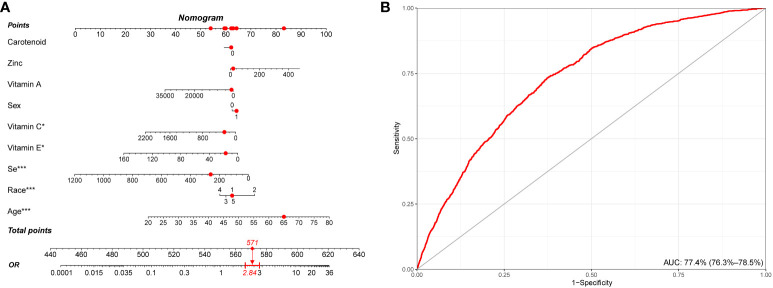
Establishment and validation of a risk prediction model for stroke. **(A)** A nomogram model based on age, race/ethnicity, and CDAI components. **(B)** ROC curve for evaluating the predictive power for stroke of the nomogram model. CDAI, composite dietary antioxidant index; ROC, receiver operating characteristic. * P value<0.05, ** P value<0.01, *** P value<0.001.

We conducted a retrospective analysis of a large cohort of participants, and the nomogram model was developed based on the most important demographical characteristics and the intake of components of CDAI for predicting stroke. Among the various components, vitamin C, vitamin E, selenium, age, and rase, showed the strongest association with stroke risk, while other components demonstrated varying degrees of influence on the stroke prediction model. This nomogram depicts a 65-year-old white man with a CDAI score of 0.5. According to our model, he exhibited a 2.84-fold higher risk of stroke (**Figure 6A**). Information from the NHANES database reveals that he does indeed have a history of stroke. The discrimination ability of the nomogram was evaluated using receiver operating characteristic (ROC) curve analysis, and the area under the curve (AUC) was found to be 77.4% (95% CI: 76.3% - 78.5%). This indicates that the model has a good ability to distinguish between stroke and non-stroke individuals based on their CDAI component intake (**Figure 6B**).

### Sensitive analysis on the association of CDAI and the prevalence of stroke using unweight logistic regression

To further validate the stability of results in the present study, we carried out sensitive analysis using unweight logistic regression. The sensitivity analysis revealed a statistically significant association between CDAI and the prevalence of stroke. Higher CDAI scores were found to be positively correlated with a decreased prevalence of stroke. The OR for stroke prevalence per unit increase in CDAI was estimated to be 0.96 (95% CI: 0.94-0.97) (**Table 4**). The unweighted logistic regression model used for the sensitivity analysis showed robustness of our results, indicating that the association between CDAI and stroke prevalence remained consistent with the main analysis.

**Table 4:** Unweighted logistic regression analysis on the association between CDAI and stroke in sensitive analysis.

	Non-adjusted model		Model I		Model II	
	OR [95% CI]	*P* value	OR [95% CI]	*P* value	OR [95% CI]	*P* value
**Continuous CDAI**	0.92 [0.91, 0.94]	<0.001***	0.94 [0.93, 0.96]	<0.001***	0.97 [0.94, 0.98]	<0.001***
**CDAI-Q1**	Reference	–	Reference	–	Reference	–
**CDAI-Q2**	0.73 [0.63, 0.84]	<0.001***	0.77 [0.67, 0.88]	<0.001***	0.92 [0.81, 1.03]	0.002**
**CDAI-Q3**	0.56 [0.48, 0.65]	<0.001***	0.63 [0.54, 0.73]	<0.001***	0.75 [0.66, 0.88]	<0.001***
**CDAI-Q4**	0.45 [0.39, 0.53]	<0.001***	0.57 [0.49, 0.67]	<0.001***	0.72 [0.64, 0.82]	<0.001***

Data are presented as OR (95% CI). Model I adjusted for age, sex, and race/ethnicity. Model II adjusted for age, sex, race, education levels, smoking, drinking, energy intake, body mass index, physical activity, hypertension, DM. *** P value<0.001, ** P value<0.01, * P value<0.05.

## Discussion

There is a growing body of evidence suggesting that oxidative stress plays a crucial role in the development and progression of stroke. Previous studies have showed that oxidative stress can lead to endothelial dysfunction and impaired blood flow regulation. This contributes to the development of vascular risk factors, such as atherosclerosis and hypertension, which are linked to an increased risk of stroke (21). Oxidative stress also activates inflammatory processes, the release of pro-inflammatory cytokines and chemokines further amplifies the damage to brain tissue, leading to a vicious cycle of inflammation and oxidative stress (22). Recently, Mu et al. conducted a whole-genomic analysis and identified six critical genes (STAT3, FPR1, AQP9, SELL, MMP9, and IRAK3) that are associated with oxidative stress and neutrophil response in early ischemic stroke. These findings may contribute to a better understanding of the pathophysiological mechanisms of stroke and potentially lead to the development of novel diagnostic biomarkers and therapeutic strategies for this serious health condition (23). Moreover, Kaur et al. investigated the potential of the neurohormone melatonin to reduce pain in central post-stroke pain (CPSP), a severe and persistent condition affecting 12% of stroke survivors. Using a rat model with thalamic lesions to mimic CPSP, they administered melatonin at different doses and performed behavioral tests and biochemical assessments. The results showed that melatonin receptors were abundant in specific brain regions. Thalamic lesions induced pain behaviors and caused mitochondrial dysfunction, oxidative stress, and neuroinflammation. However, melatonin treatment dose-dependently reversed these effects, especially the oxidative stress condition to improve pain behaviors and protect against neuronal damage (24).

Diet plays a crucial role in regulating the body’s oxidative stress levels. Vitamin C and vitamin E are two essential antioxidant vitamins that can scavenge and neutralize free radicals, thus reducing the extent of oxidative stress (25, 26). Polyphenols are a group of natural antioxidant compounds found in fruits, vegetables, tea, and nuts. They can also contribute to lowering oxidative stress levels (27). Additionally, some minerals like zinc, selenium, and copper are also vital components of antioxidants, playing a key role in enzyme activity (28–30). Western diet typically refers to the dietary habits of typical Western countries, characterized by high-fat, high-sugar, and high-salt intake. There is a close relationship between this dietary pattern and the levels of oxidative stress in the body. Excessive fat intake can lead to lipid peroxidation, resulting in the generation of a large number of reactive oxygen species (ROS). High-sugar diet can trigger insulin resistance, increasing inflammatory response and oxidative stress. High-salt diet can raise blood pressure, damage vascular endothelial cells, and further elevate oxidative stress levels. The Mediterranean diet is a traditional dietary pattern characteristic of the Mediterranean region. Zhou et al. also conducted a cross-sectional study and found that increased dietary inflammation index associated with higher prevalence rate of hypertension, and dietary inflammation index was also associated with increased prevalence of coronary heart disease (31, 32). It emphasizes fresh fruits, vegetables, whole grains, olive oil, nuts, seeds, fish, and limited amounts of red meat and dairy products. The Mediterranean diet is rich in antioxidants, Omega-3 fatty acids, and polyphenols, and it has anti-inflammatory properties, which help reduce oxidative stress levels in the body (33–35).

CDAI takes into account the dietary intake of various antioxidants, such as vitamins (vitamin C, vitamin E), minerals (zinc, selenium), and phytochemicals (flavonoids, carotenoids) that are known to have antioxidant properties (36–38). It assigns specific weights to these dietary components based on their potency as antioxidants. Individuals are then scored based on their daily intake of foods rich in antioxidants. The higher the intake of antioxidant-rich foods, the higher the CDAI score, indicating a diet with better antioxidant capacity. The CDAI is used in nutritional research to study the relationship between dietary antioxidant intake and oxidative stress-related health outcomes. Zhao et al. investigated the relationship between the CDAI and depression using data from the NHANES database, and their results showed a negative non-linear association between CDAI and depression, with 0.16 being the inflection point. Before the inflection point, each unit increase in CDAI was associated with a 30% decrease in the risk of depression, and after the inflection point, the risk of depression was reduced by 11% for each unit increase. The study suggests that an antioxidant-rich diet may be a protective factor against depression, but further research is needed to better understand their association (39). Another perspective study aimed to investigate the correlation between CDAI and all-cause and cardiovascular mortality risk by enrolling 44,031 participants. Results showed a linear relationship between CDAI and all-cause mortality, with higher CDAI associated with reduced risk. The study also found a similar trend for cardiovascular mortality. The findings suggest that an antioxidant-rich diet may significantly lower the risk of cardiovascular mortality (40). Wu et al. aimed to investigate the association between the CDAI and handgrip strength in 6,019 American adults. They found a positive correlation between CDAI and handgrip strength, but this association varied by gender. In males, CDAI was significantly associated with handgrip strength, and dietary intake of vitamin E, zinc, and selenium showed positive correlations with handgrip strength. However, in females, the association between CDAI and handgrip strength was not significant, and only dietary zinc intake was positively correlated with handgrip strength (41). In the present study, we studied the association of CDAI with the prevalence of stroke in a large population. Higher intake of antioxidant-rich food can significantly reduce the prevalence of stroke. Moreover, we conducted RCS analysis to explore the relationships between the CDAI and its individual components with the prevalence of stroke. The RCS analysis revealed a significant association between CDAI and stroke prevalence, with a notable non-linear trend. As CDAI scores increased, the odds of stroke exhibited a gradual decline up to a certain threshold. Beyond the inflection point, the protective effect seemed to stabilize, suggesting a potential saturation effect at higher CDAI levels. Subgroup analysis and sensitive analysis were also conducted to verify the stability of the conclusion drawn in the present study. Increasing the dietary antioxidant levels can help protect brain cells and blood vessels, reducing the risk of stroke.

There are some implications of this study. First of all, the study highlights the potential importance of dietary antioxidants in reducing the prevalence of stroke. Promoting a diet rich in antioxidant-containing foods, such as fruits, vegetables, nuts, and olive oil, could be beneficial for stroke prevention. The findings provide a basis for further research on the role of dietary antioxidants in stroke prevention and management. Longitudinal studies and randomized controlled trials are needed to establish causality and explore the potential therapeutic effects of antioxidant-rich diets. Moreover, public health initiatives may consider emphasizing the consumption of antioxidant-rich foods to promote brain health and reduce stroke risk in the population. Thirdly, healthcare professionals could consider providing dietary recommendations that focus on increasing antioxidant intake to patients at risk of stroke or those with a history of stroke. There are also some limitations of this study. Firstly, we used an observational design, which means that it can only establish associations and not causation. Other confounding factors or variables not accounted for in the analysis could influence the observed relationships. Secondly, dietary information in NHANES is often self-reported, which may introduce recall bias or misclassification of dietary intake. People might not accurately remember or report their actual food consumption, leading to potential inaccuracies in the assessment of the CDAI. We used cross-sectional data, which captures data at a single point in time. As a result, it cannot establish the temporal relationship between dietary antioxidant intake and stroke prevalence or evaluate cause-and-effect associations. Additionally, certain indicators highly correlated with the occurrence of stroke, such as macronutrients and the intake of fruits, vegetables, saturated and unsaturated fatty acids, and cholesterol, were not included in our study. This is also recognized as a limitation in our manuscript. Lastly, due to the nonlinear relationship between CDAI and stroke risk, the stroke risk prediction model based on CDAI in this study may suffer from overfitting. We did not validate the ridge plot model derived in this study using external data. We hope to address this in future work by further validating the generalizability of the data in external datasets.

## Conclusion

Our study highlights a significant association between the CDAI and stroke prevalence, indicating that a diet rich in antioxidants may play a crucial role in reducing the risk of stroke. These findings from the NHANES data spanning from 1999 to 2018 suggest that promoting dietary choices high in antioxidants could potentially be an important preventive strategy for stroke. Further research and interventions focusing on enhancing antioxidant intake may offer valuable insights into stroke prevention and management.

## Data availability statement

The original contributions presented in the study are included in the article/**Supplementary Material**. Further inquiries can be directed to the corresponding author.

## Ethics statement

This study was approved by National Center for Health Statistics Research Ethics Review Board. The participants provided informed consent to participate in the NHANES survey. The NHANES protocol complies with the U.S. Department of Health and Human Services Policy for the Protection of Human Research Subjects. NCHS IRB/ERC Protocol number: 2011-17. Ethical review and approval were waived for this study as it solely used publicly available data for research and publication. The studies were conducted in accordance with the local legislation and institutional requirements. The participants provided their written informed consent to participate in this study.

## Author contributions

T-QT: Conceptualization, Data curation, Investigation, Software, Validation, Writing – original draft, Writing – review & editing. JL: Software, Validation, Writing – review & editing. F-FH: Data curation, Formal analysis, Writing – original draft. Q-QL: Writing – review & editing. Z-ZH: Visualization, Writing – review & editing. YS: Conceptualization, Supervision, Writing – original draft.
