# Supplementary material for: Association of composite dietary antioxidant index with prevalence of stroke: insights from NHANES 1999-2018
Source: Front Immunol. 2024 Mar 8;15:1306059. doi: 10.3389/fimmu.2024.1306059 (PMC10957548; doi:10.3389/fimmu.2024.1306059)
Supplement: Supplementary file 1 [file Table_1.docx]

**Table S1. Clinical Characteristics of the Study Population Grouped by CDAI quantiles.**

| Variables | CDAI-Q1 | CDAI-Q2 | CDAI-Q3 | CDAI-Q4 | *P* value |
| --- | --- | --- | --- | --- | --- |
| Age, years |  |  |  |  | <0.001*** |
| 18-40 years | 39.65 [38.18, 41.12] | 38.80 [37.51, 40.10] | 38.54 [36.86, 40.21] | 42.15 [40.63, 43.67] |  |
| 40-60 years | 38.02 [36.66, 39.39] | 38.68 [37.41, 39.96] | 40.68 [39.20, 42.15] | 40.76 [39.42, 42.10] |  |
| > 60 years | 22.32 [21.28, 23.37] | 22.51 [21.31, 23.72] | 20.79 [19.67, 21.91] | 17.09 [15.88, 18.31] |  |
| Sex-male, % | 43.36 [42.32, 44.41] | 49.46 [48.27, 50.65] | 50.37 [49.11, 51.64] | 52.94 [51.76, 54.12] | <0.001*** |
| Race, % |  |  |  |  | <0.001*** |
| Non-Hispanic White | 63.51 [60.71, 66.31] | 68.03 [65.61, 70.45] | 70.45 [68.23, 72.67] | 69.79 [67.69, 71.89] |  |
| Non-Hispanic Black | 15.17 [13.48, 16.85] | 11.09 [9.83, 12.35] | 9.43 [8.31, 10.56] | 9.89 [8.79, 10.99] |  |
| Mexican American | 8.03 [6.78, 9.28] | 8.59 [7.36, 9.82] | 8.21 [7.05, 9.38] | 8.29 [7.09, 9.49] |  |
| Other Hispanic | 6.36 [5.20, 7.53] | 5.41 [4.49, 6.34] | 5.19 [4.42, 5.96] | 4.96 [4.26, 5.66] |  |
| Other | 6.93 [6.14, 7.73] | 6.87 [6.08, 7.66] | 6.71 [5.99, 7.43] | 7.07 [6.30, 7.84] |  |
| Smoking, % | 30.84 [29.50, 32.19] | 22.26 [21.01, 23.51] | 19.01 [17.90, 20.13] | 17.54 [16.46, 18.63] | <0.001*** |
| Drinking, % | 86.80 [85.69, 87.90] | 89.14 [88.16, 90.12] | 89.42 [88.12, 90.71] | 91.38 [90.29, 92.46] | <0.001*** |
| Education level, % |  |  |  |  | <0.001*** |
| Below high school | 7.69 [6.97, 8.41] | 5.58 [4.94, 6.21] | 4.38 [3.93, 4.83] | 3.71 [3.22, 4.19] |  |
| High school | 43.44 [41.85, 45.02] | 36.00 [34.32, 37.68] | 31.25 [29.71, 32.79] | 29.17 [27.74, 30.59] |  |
| Above high school | 48.87 [47.23, 50.52] | 58.42 [56.60, 60.25] | 64.38 [62.70, 66.05] | 67.13 [65.51, 68.74] |  |
| SBP, mmHg | 122.55 [122.06, 123.03] | 121.90 [121.42, 122.38] | 121.36 [120.89, 121.83] | 120.41 [119.97, 120.85] | <0.001*** |
| DBP, mmHg | 71.28 [70.91, 71.65] | 71.33 [70.90, 71.76] | 71.53 [71.15, 71.91] | 71.76 [71.38, 72.14] | 0.11 |
| DM, % | 5.90 [5.84, 5.96] | 5.85 [5.79, 5.90] | 5.88 [5.80, 5.95] | 5.81 [5.76, 5.87] | 0.19 |
| FBG, mmol/L | 5.62 [5.59, 5.64] | 5.60 [5.58, 5.63] | 5.58 [5.56, 5.61] | 5.53 [5.50, 5.55] | <0.001*** |
| HbA1c, % | 94.43 [93.74, 95.11] | 94.78 [94.09, 95.46] | 95.31 [94.62, 96.00] | 96.40 [95.70, 97.10] | <0.001*** |
| eGFR, ml/min/1.73m^2^ | 14.46 [13.41, 15.50] | 13.44 [12.67, 14.21] | 12.20 [11.34, 13.06] | 10.96 [10.15, 11.76] | <0.001*** |
| TG, mmol/L | 1.50 [1.45, 1.55] | 1.50 [1.45, 1.55] | 1.49 [1.42, 1.55] | 1.47 [1.43, 1.51] | 0.74 |
| TC, mmol/L | 5.10 [5.06, 5.13] | 5.07 [5.05, 5.10] | 5.07 [5.04, 5.10] | 5.02 [4.99, 5.06] | 0.01* |
| LDL-C, mmol/L | 3.01 [2.97, 3.05] | 3.03 [3.00, 3.07] | 2.97 [2.94, 3.00] | 2.94 [2.90, 2.97] | <0.001** |
| HDL-C, mmol/L | 1.36 [1.35, 1.37] | 1.36 [1.35, 1.37] | 1.38 [1.36, 1.39] | 1.39 [1.38, 1.40] | 0.005** |
| RBC, ×10^9^/L | 4.70 [4.68, 4.71] | 4.73 [4.71, 4.74] | 4.73 [4.71, 4.74] | 4.75 [4.73, 4.76] | <0.001*** |
| WBC, ×10^9^/L | 7.46 [7.39, 7.54] | 7.33 [7.26, 7.40] | 7.17 [7.11, 7.24] | 7.10 [7.04, 7.17] | <0.001*** |
| NE, ×10^9^/L | 4.45 [4.39, 4.50] | 4.35 [4.30, 4.40] | 4.23 [4.18, 4.28] | 4.20 [4.15, 4.25] | <0.001*** |
| Monocyte, ×10^9^/L | 0.56 [0.56, 0.57] | 0.57 [0.56, 0.57] | 0.56 [0.55, 0.56] | 0.55 [0.55, 0.56] | 0.002** |
| LY, ×10^9^/L | 2.20 [2.18, 2.22] | 2.17 [2.14, 2.20] | 2.14 [2.12, 2.16] | 2.11 [2.08, 2.14] | <0.001*** |
| PLT, ×10^6^/L | 260.33 [258.21, 262.45] | 254.82 [252.86, 256.78] | 251.95 [249.83, 254.08] | 250.10 [248.22, 251.99] | <0.001*** |
| Hemoglobin, g/L | 14.23 [14.17, 14.30] | 14.35 [14.30, 14.40] | 14.37 [14.32, 14.42] | 14.44 [14.39, 14.49] | <0.001*** |
| CHD, % | 3.86 [3.37, 4.36] | 3.59 [3.10, 4.08] | 2.63 [2.22, 3.04] | 2.36 [1.95, 2.77] | <0.001*** |
| Angina, % | 2.87 [2.39, 3.36] | 2.20 [1.86, 2.53] | 1.98 [1.58, 2.37] | 1.66 [1.33, 1.99] | <0.001*** |
| HF, % | 3.10 [2.70, 3.50] | 2.23 [1.86, 2.59] | 1.58 [1.28, 1.88] | 1.38 [1.10, 1.66] | <0.001*** |
| Hypertension, % | 39.23 [37.88, 40.58] | 37.91 [36.60, 39.21] | 35.28 [33.90, 36.66] | 33.70 [32.27, 35.13] | <0.001*** |
| Heart attack, % | 4.31 [3.76, 4.87] | 3.36 [2.96, 3.75] | 2.87 [2.40, 3.35] | 2.10 [1.73, 2.46] | <0.001*** |

Continuous variables are presented as the mean [95% CI], category variables are presented as the proportion [95% CI]. CI, confidence interval; SBP, systolic blood pressure; DBP, diastolic blood pressure; DM, diabetes; FBG, fasting blood glucose; HbA1c, glycated hemoglobin; eGFR, estimated glomerular filtration rate; BMI, body mass index; WC, waist circumference; TG, triglycerides; TC, total cholesterol; LDL-C, low-density lipoprotein cholesterol; HDL-C, high-density lipoprotein cholesterol; RBC, red blood cells; WBC, white blood cells; NE, neutrophils; LY, lymphocytes; PLT, platelets; CHD, coronary artery disease; HF, heart failure. * *P* value<0.05, ** *P* value<0.01, *** *P* value<0.001.
